# Pesticide threshold weighing indicator: application in the State of Paraná, Brazil

**DOI:** 10.1590/1980-549720250045

**Published:** 2025-08-08

**Authors:** Viviane Serra Melanda, Humberto Cereser Ibañez, Henrique Aparecido Laureano, Luíza Siqueira Lima, Bonald Cavalcante Figueiredo, Cláudia Sirlene Oliveira

**Affiliations:** I Instituto de Pesquisa Pelé Pequeno Príncipe - Curitiba (PR), Brazil.; II Faculdades Pequeno Príncipe - Curitiba (PR), Brazil.; III Secretaria de Estado da Saúde do Paraná - Curitiba (PR), Brazil.

**Keywords:** Health status indicators, Agrochemicals, Epidemiology, Water pollutants

## Abstract

**Objective:**

To analyze data from the Information System for Monitoring the Quality of Water for Human Consumption (Sisagua) to develop an environmental health indicator that assesses the risk of pesticide residues in drinking water.

**Methods:**

This is an ecological epidemiological study using retrospective data. Information on the Limit of Detection (LOD), Limit of Quantification (LOQ), and Maximum Permissible Value (MPV) of pesticide residues in drinking water, recorded by municipalities in the state of Paraná between 2014 and 2020, was analyzed. Descriptive and inferential statistical approaches were employed, including tests of association, correlation, and hypothesis testing, as well as probability analysis, through spatial and temporal analyses.

**Results:**

A Pesticide Threshold Weighting Indicator (iPLA) was developed, with an explanatory capacity of variability greater than 77%. The risk-attributable variable was mainly related to the MPV, which received the highest weighting, while the LOD and LOQ were assigned lower weights.

**Conclusion:**

The iPLA demonstrated the ability to represent pesticide concentration levels in drinking water. The risk categories defined by the indicator - controlled, silent, and alert - represent a highly useful tool for public health surveillance, as they enable the identification of local drinking water risk levels to human health. Moreover, the iPLA supports public management in implementing control actions and improvements in the quality of water for human consumption.

## INTRODUCTION

The risk assessment of pesticides is fundamental, as they are associated with adverse effects on vital organic functions^1^ ^,^ ^2^ ^,^ ^3^ ^,^ ^4^ ^,^ ^5^. Although in Brazil water potability standards had been officially based on MS/GM Ordinance No. 635/1975^6^, only in 2011, with the publication of MS/GM Ordinance No. 2,914/2011, the procedures for control and surveillance of water quality for human consumption were instituted. The MS/GM Ordinance No. 2,914/2011 establishes quality standards for water potability, indicating chemical substances that pose risk to health. These substances are grouped into four categories: inorganic, organic, pesticides and disinfectants, and secondary disinfection products^7^. These standards were updated in 2021, with the publication of MS/GM Ordinance No. 888^8^.

Concomitantly, the Information System for Monitoring the Quality of Water for Human Consumption (*Sistema de Informação da Vigilância da Qualidade da Água para Consumo Humano* - Sisagua), the official information system provided by the Brazilian Ministry of Health, was updated, aiming at better control of inconsistencies in data entry as well as architectural improvements to facilitate its use^9^. Sisagua is part of the National Surveillance Program of Water Quality for Human Consumption (*Programa Nacional de Vigilância da Qualidade da Água para Consumo Humano* - Vigiagua), which assists in the management of health risks associated with the quality of water for human consumption^10^. The municipalities are responsible for feeding Sisagua by recording the results obtained from the water potability analyses.

Despite the importance of pesticide exposure patterns defined by regulatory agencies, there are technical barriers regarding the reference values used in the validation of analytical methods^11^. The existence of such difficulties can harm or delay the recognition of health risk patterns of the population exposed to pesticides.

Environmental health indicators can subsidize prevention, risk control, and health promotion strategies, as well as monitoring epidemiological outcomes of this relationship^12^; however, we did not identify an indicator model in the scientific literature that points to the relationship between the presence of pesticide residue in drinking water and the potential risk to human health.

In order to bring knowledge of the risks for human health arising from the presence of pesticide residues in drinking water, in this study, we developed a pesticide threshold weighting indicator (from Portuguese, *indicador de ponderação de limiar de agrotóxicos* - iPLA). The objective was to simplify the monitoring and classification of risks, enabling more agility in the development of health policies and intersectoral strategies involving the environment and public health.

## METHODS

This is a cross-sectional epidemiological study, based on retrospective data, with descriptive and inferential statistical analysis. Inferential analyses include association, correlation, and hypothesis testing, in addition to probability evaluation through spatial and temporal analyses. For statistical analyses, the R^13^ and Geomedicina^14^ software were used. This study was approved by the Research Ethics Committee of Faculdades Pequeno Príncipe (Certificate of Presentation for Ethical Consideration [CAAE] No. 29734420.9.0000.5580), as well as by the Research Ethics Committee of the State Department of Health of Paraná (CAAE No. 4.000.930/29734420.9.3002.5225), based at Hospital do Trabalhador.

An indicator was developed to assess the risk of the presence of pesticide residues in drinking water. To this end, secondary data were used from all available records in Sisagua referring to the state of Paraná (Brazil), in the historical series from 2014 to 2020. Sisagua samples are collected in the municipalities, in surface waters distributed by water supply systems and/or collective alternative solutions^15^.

These are public domain data, made available by the Ministry of Health and directly obtained via download in the virtual environment of the federal government, in the open dataset of Sisagua, option “Controle Semestral” [Semester Control]. All the municipalities that presented at least one record in the group of pesticide parameters were included in the analysis.

When processing the database, it was observed that the results contained both numerical (in mg/L or μg/L) and nominal (LOWER_LOD or LOWER_LOQ) records. Significant variations were identified in the record fields of limits of detection (LOD) and quantification (LOQ) of each water treatment plant. To integrate nominal and numerical data, these quantification records were counted, in such a way that both numerical and nominal results could be deemed equivalent.

The nominal values were assigned to the following classes:

- Lower than the LOD (<LOD);
- Lower than the LOQ (<LOQ).

The numerical values were assigned to the following classes:

- Between LOQ and the Maximum Permissible Value [MPV] (≥LOQ and <MPV);
- Higher than or equal to the MPV (≥MPV).

Thus, after accounting for all occurrences recorded in the municipalities in one of the four ranges, the weight count obtained from the principal component analysis (PCA) was applied, which corroborated the identification of the optimal linear combination of the variables that best explain the variability of the data.

The elimination of inconsistencies in the values recorded in Sisagua was carried out as follows:

- Numerical values equal to 0 were assigned to class <LOD;
- Among the numerical values higher than the MPV, records whose Z*-*score [(recorded value - mean of all values)/standard deviation] was higher than three times the MPV were excluded.

### Data availability statement

All datasets were generated or analyzed in the current study.

## RESULTS

### Development of the Pesticide Threshold Weighting Indicator

This process involved reading and circumstantial interpretation of the data, considering the following evaluations:

- Evaluation 1: variables’ design.

- <LOD;
- <LOQ;
- <MPV;
- ≥MPV.

- Evaluation 2: assigning weights to variables.

**Figure 1.**
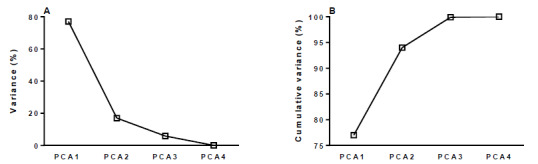
Analysis of percentage variance and cumulative variance, by principal component of pesticides in drinking water in the municipalities of Paraná, 2014-2020.

The weight of each variable was determined by PCA, considering the measurements of LOD, LOQ, and MPV as independent and potentially correlated in space (Table 1). For better variability accommodation, the results were normalized, allowing us to verify that the four components, together, explained 100% of the variability of the measures. We observed that PCA1 (≥MPV) explained 77% of the total variability, while PCA2, PCA3, and PCA4 together represented the remaining 23% (Figure 1). As the first principal component (PCA1) explained 77% of the total variability, it was selected for developing the PCA indicator, ensuring the preservation of most relevant information. Taking this into consideration, we carried out a comparative study of statistical variability to verify the correspondence between the principal components and the results obtained from the samples (Table 2).

**Table 1.** Variability of the number of records by the Limit of Detection, Limit of Quantification, and Maximum Permissible Value vectors, Paraná, 2014-2020.

Indicator	<LOD	<LOQ	<MPV	>MPV
First eigenvector (weights)	0.16021	0.98707	0.00247	-0.00179
PCA (No. of records)	0.07	0.71	1.43	2.14

LOD: Limit of Detection; LOQ: Limit of Quantification; MPV: Maximum Permissible Value; PCA: Principal Component Analysis.

**Table 2.** Descriptive measures of the distribution of the analyzed variables regarding the results of the records of pesticides in drinking water in the municipalities of Paraná, 2014-2020.

	>LOD	>LOQ	>MPV	≥MPV
Minimum	0	0	0	0
First quartile	80	214	1	0
Median	109	312	10	0
Mean	169.85	462.52	46.84	10.26
Third quartile	190.5	566.0	46.0	6.5
Maximum	3937	5344	2382	225
Standard deviation	251.06	507.81	142.14	24.99
Moran’s	0.0110367	0.110101343	0.0634899	0.1443155
Expected value	-0.0025216	-0.0025126	-0.0025126	-0.0025156
Variance	0.0005701	0.0009257	0.0007488	0.0009084
p-value	0.570383	0.000214	0.015862	0.000011

LOD: Limit of Detection; LOQ: Limit of Quantification; MPV: Maximum Permissible Value.

PCA: Principal Component Analysis

The robustness of the model was indirectly evaluated by the proportion of variability explained by PCA1, indicating its adequacy to represent the data without the need for additional components. Moreover, as PCA is an unsupervised method, its traditional validation by techniques, such as cross-validation, does not directly apply to it, considering that there is no set of training and testing to be compared^16^. Nevertheless, we verify the stability of factor loads (weights) and their interpretative coherence, ensuring that the development of the indicator is consistent with the data structure.

- Evaluation 3: developing the iPLA.

As the number of records may vary in each time series, the pesticide threshold weighting indicator (iPLA) was developed aiming at enabling the comparability of new scenarios. To assign greater weight to the result counts of drinking water analyses that fit the defined ranges in relation to the MPV (>LOQ and <MPV; and ≥MPV), the following constants were applied, based on the PCA values (Table 1):

- For results in the ≤LOD range: 0.07;
- For results in the >LOD and ≤LOQ range: 0.71;
- For results in the >LOQ and <MPV range: 1.43;
- For results in the ≥MPV range: 2.14.

The iPLA calculation is represented by Equation 1:

iPLA = lower_lod/tn_result*0.07 + lower_loq/tn_result*0.71 + lower_mpv/tn_result*1.43 + maigu_mpv/tn_result*2.14 (1)

Where:

lower_lod: count of the results of analysis of a certain pesticide in the water treatment plants of a given municipality, whose values were ≤LOD;
tn_result: total number of results related to a certain pesticide obtained from the water treatment plants of a given municipality during the analyzed period;
lower_loq: count of the results of analysis of a certain pesticide in the water treatment plants of a given municipality, whose values were >LOD and ≤LOQ;
lower_mpv: count of the results of analysis of a certain pesticide in the water treatment plants of a given municipality, whose values were >LOQ and <MPV;
maigu_mpv: count of the results of analysis of a certain pesticide in the water treatment plants of a given municipality, whose values were ≥MPV;
0.07: the constant applied to the count of results with values ≤LOD;
0.71: the constant applied to the count of results with values >LOD and ≤LOQ;
1.43: the constant applied to the count of results with values >LOQ and <MPV;
2.14: the constant applied to the count of results with values ≥MPV.

Therefore, the weighting of pesticide thresholds established constants that assigned greater weight to the municipality indicator in cases in which the results were close to (constant: 1.43) or higher than (constant: 2.14) the MPV. Conversely, when the results predominated between the limits of detection and quantification, the assigned constants - respectively, 0.07 and 0.71 - assigned lower weight to the indicator.

The model adopted in the development of iPLA validated the proposal of the environmental health indicator for the analysis of pesticide concentration in drinking water, as it presented statistically-proven and representative results of the reality. To interpret the results of iPLA, three groups were defined based on the variations concerning the LOD, LOQ, and MPV categories, which received classifications at risk levels (Table 3). The definition of the risk level assigned to each group was established based on the thresholds of the variables related to the respective constants. Thus, iPLA enabled an objective interpretation of the risk associated with the presence of pesticides in water destined for human consumption.

- Evaluation 4: PCA versus iPLA.

By comparing the descriptive measures of iPLA and PCA, we observed that iPLA presented excellent capacity to represent drinking water contamination. It had a lower standard deviation than that of the PCA indicator and used dynamic weights, that is, that varied according to the number of records, enabling the equivalence of the obtained results when the indicator was used in different sites (Table 4).

**Table 3.** Risk categories of the Pesticide Threshold Weighting Indicator in drinking water.

Category	iPLA variation	Interpretation according to count percentages in classes <LOD, <LOQ, ≥LOQ and <MPV and ≥MPV
Silent (I)	≥0 and <0.7	Location that does not present pesticide detection and requires investigation regarding the quality of chemical analyses of samples and data entered in Sisagua.
Controlled (II)	≥0.7 and <1	Location that presents detection of pesticide residues whose values are not quantifiable and, therefore, are of unqualified risk.
Attention (III)	≥1	Location that presents detection of pesticide residues whose values qualify for the existence of risk to human health.

LOD: Limit of Detection; LOQ: Limit of Quantification; MPV: Maximum Permissible Value; iPLA: Pesticide threshold weighting indicator; Sisagua: Information System for Monitoring the Quality of Water for Human Consumption.

**Table 4.** Descriptive analysis of the indicators of pesticides in drinking water, Principal Component Analysis and Pesticide Threshold Weighting Indicator.

Indicator	Minimum	First quartile	Median	Mean	Third quartile	Maximum	Standard deviation
iPLA	0	0.53	0.58	0.62	0.68	1.51	0.2
PCA	-0.94	-0.5	-0.29	0	0.21	9.44	1

iPLA: Pesticide Threshold Weighting Indicator; PCA: Principal Component Analysis.

#### Application of the pesticide threshold weighting indicator for situational epidemiological risk analysis in Paraná

For spatial epidemiological analysis, we chose to apply the five-year historical series (2014-2018) in the state of Paraná to verify trends in the obtained results. 27 types of pesticide residues were found in drinking water samples, according to Sisagua records, in 393 of the 399 municipalities of Paraná (Supplementary Table 1: https://drive.google.com/drive/folders/1bTyymbYahQXyZKlsn7felApqtyyrJneV?usp=sharing). In Table 5, we show the frequency and percentage of the analyses recorded in Sisagua between 2014-2018, considering the LOD, LOQ, and MPV variables. The counts cover the 27 pesticides analyzed in this study, totaling 176,533 records. Of these, the majority (82.2%) corresponds to nominal values <LOD.

**Table 5.** Frequency and percentage of water analyses recorded in Sisagua for the variables Limit of Detection, Limit of Quantification, and Maximum Permissible Values in the state of Paraná (2014-2018).

Year	Number of records <LOD (%)	Number of records <LOQ (%)	Number of records ≥LOQ <MPV (%)	Number of records ≥MPV (%)	Total number of records
2014	2,567 (11.05)	19,663 (84.65)	625 (2.69)	374 (1.61)	23,229
2015	3,442 (10.90)	27,100 (85.82)	668 (2.12)	368 (1.17)	31,578
2016	2,512 (7.00)	32,090 (89.42)	806 (2.25)	478 (3.91)	35,886
2017	5,556 (13.05)	31,713 (74.48)	3,648 (8.57)	1,664 (3.91)	42,581
2018	1,830 (4.23)	34,547 (79.86)	5,872 (13.57)	1,010 (2.33)	43,259

LOD: Limit of Detection; LOQ: Limit of Quantification; MPV: Maximum Permissible Value; Sisagua: Information System for Monitoring the Quality of Water for Human Consumption.

**Figure 2.**
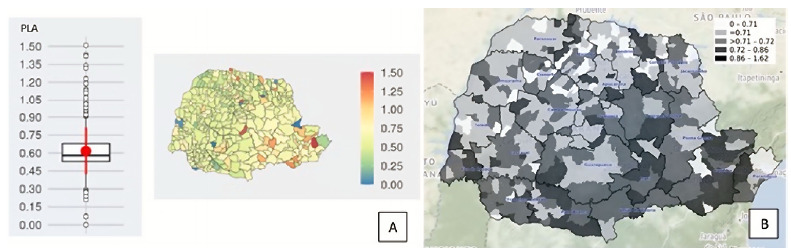
Level of risk of pesticides in drinking water recorded by iPLA, by (a) Municipalities and (b) Population density, Paraná, 2014-2018.

In many municipalities in the state of Paraná, we verified results of pesticide concentration in drinking water samples with <LOQ values, and some recorded samples with concentrations above the permissible values. In addition, there was an increase in the number of records with ≥MPV values over the years. The risk associated with the level of iPLA is especially high in municipalities in the southeastern region of Paraná, especially Vale do Ribeira, where there are municipalities near the state of São Paulo and which are classified as level III of attention. (Figure 2A).

iPLA: Pesticide Threshold Weighting Indicator.

When investigating whether there is a correlation between productivity (kg/ha) in Paraná, obtained from the Monitoring System of Trade and Use of Pesticides (*Sistema de Monitoramento do Comércio e Uso de Agrotóxicos* - Siagro), and iPLA, we found no correlation between these indicators according to the results (Supplementary Figure 1: https://drive.google.com/drive/folders/1bTyymbYahQXyZKlsn7felApqtyyrJneV?usp=sharing)). In turn, according to the analysis of the geographic distribution of the risk of pesticides (iPLA) by population density, we verified that the southern, southeast, and southwest regions of the state of Paraná stand out with the highest rates (Figure 2B). According to iPLA, most municipalities in Paraná present level-III risk, which indicates an unsafe health condition associated with the presence of pesticides in drinking water.

## DISCUSSION

In 1998, the World Health Organization (WHO) established criteria for the development of indicators in environmental health: general applicability, scientific soundness, and usefulness for users^17^ ^,^ ^18^. The iPLA was developed aiming at overcoming the barrier of applicability to the user, being structured to facilitate the interpretation of results through risk categories. We demonstrated its general applicability, considering that it was built based on results of drinking water samples analyzed by municipal supply networks. The scientific soundness of iPLA was also evidenced by statistical analysis, according to which we identified significant validity in its capacity to represent the degree of contamination by pesticides in drinking water. Regarding the purposes described by the WHO on the use of indicators for health monitoring^19^, iPLA is justified by its ability to describe and evaluate risks, as well as to promote accountability, by highlighting the susceptibility of the exposed population and evidencing the real quality of drinking water available to the population.

In a recent publication in Europe, the importance of monitoring pesticides in surface water, in absolute numbers, was verified, aiming at formulating quality standards for groundwater. Researchers observed differences in monitoring records such as the number of samples, monitored pesticides, and analyzed locations. These differences may compromise the interpretation of trends in the continent^20^. In our study, iPLA was developed based on data recorded in the database of a national and official information system of Brazil, Sisagua, and can therefore be reproduced in all administrative units of the country. We statistically demonstrated that iPLA has the capacity to represent water contamination by pesticides, being interpreted as a negative indicator.

Having as characteristic the search for innovation in agriculture, including new irrigation technologies for increasing productivity, Paraná stands out as a federative unit with intense investment activity and fundraising for agribusiness, significantly contributing to the growth of national wealth^21^ ^,^ ^22^. In this study, we analyzed the correlation between agricultural productivity and the concentration of pesticides in drinking water in the state of Paraná, through iPLA, and identified no correlation between these variables. Authors of a recent study, who investigated the relationship between agricultural productivity and pesticide use in Brazil, did not present results that allow us to confirm the existence of correlation between these two variables exclusively^23^. Thus, researchers who seek to analyze the correlation between the use of pesticides and productivity should consider the various elements that co-participate in productive management.

Although water potability monitoring is a widely adopted practice by governments around the world, standardization of analytical methods still poses a challenge to be overcome^11^. There is still an important gap in the existence of a health indicator capable of classifying the level of contamination of drinking water by pesticide residues that has continuously updated data, with a viable calculation, and that is easy to understand for professionals working in environmental health surveillance. iPLA is an environmental health indicator of practical application, as its monitoring is based on the frequency of records in Sisagua, gathering nominal and quantitative results, transforming them into a counting variable inserted into an equation composed of predefined and consistent constants. The obtained results are parameterized in risk categories, which makes their analysis simple and accessible.

In Brazil, the legislation allows relatively high levels of pesticides in drinking water and disregards the sum of MPV when multiple residues are present in the same sample. In addition, pesticides not covered by current regulations have no established MPV, which makes it difficult to adopt intervention measures^23^. Even when the results of the analyses comply with the established limits, it is essential to maintain health surveillance actions on potentially exposed populations. There are significant differences in MPV of pesticides, both in food and water, among the main global producers, such as Brazil, the United States, China, Japan, and also in relation to WHO guidelines. The parameters defined by regulatory standards vary between these countries. For example, in the European Union, regardless of the active ingredient, the MPV for drinking water is 0.0001 mg/L per substance, and the MPV for the sum of all substances is 0.0005 mg/L. Conversely, in Brazil, the United States, China, and Japan, each pesticide has a regulated individual MPV, without the requirement of a total maximum limit for the sum of the residues present in the same sample^24^.

Within this context, the development of iPLA is highly relevant, because it considers the records equal to or higher than the MPV, regardless of the chemical agent. This flexible characteristic is strategic in the analysis of the data used by the indicator, as iPLA remains applicable even in the face of changes in normative parameters - as occurred in Brazil with the publication of GM/MS Ordinance No. 888/2021, which modified the tolerance levels of the MPV and altered the group of allowed pesticides.

In a recent study on the presence of pesticides in water samples monitored by Vigiagua, 41,780 samples were analyzed. The most frequently quantified pesticides were: atrazine, metolachlor, glyphosate, and 2.4-D. Less than 10% of the detections exceeded the limit of quantification; however, the authors identified a high percentage of substances, although not quantifiable, which possibly reflects limitations in the analytical techniques used, and not necessarily the absence of risk^25^.

In this sense, iPLA, by pointing out the circumstantial risk to human health resulting from the exposure to pesticides, is a tool of great utility for public management. The indicator enables recognizing the level of risk to the health of the population exposed to any pesticide present in drinking water, from category-I municipalities, which are classified as silent and whose monitoring should be qualified; category-II municipalities, whose monitoring is sensitive and the results do not point to the risk to human health; and category-III municipalities, whose results point to a situation of attention and alert for health surveillance.

During the development of iPLA, we identified a relevant limitation related to the variability of the analytical methods used to measure the values of LOD, LOQ, and MPV, in addition to differences in quality control among laboratories that analyze the samples of drinking water. These factors can generate underreporting or variations in the quality and frequency of data recorded in the official information system (Sisagua), which is fed according to the technical training of the professionals in charge. The possibility of entering nominal and numerical data characterizes an accuracy fragility in relation to the parameterization of results. We observed that, in 25% of the analyzed records, the results were equal to the MPV established for the chemical agent. This statistical regularity raises the hypothesis of inconsistencies in the database, because laboratory tests, by their nature, should generate a wide variety of results, not such a significant concentration in the exact value of the permissible limit.

It is worth noting that only health surveillance control data - such as those on residual disinfectants, turbidity, color, pH, fluoride, heterotrophic bacteria, total coliforms, and *Escherichia coli* - are automatically shared. The other Sisagua data are manually entered by professionals of the municipal departments of health or by water supply utilities for human consumption^26^. Thus, we highlight the importance of critical analysis and continuous qualification of data entered in the system.

Despite these limitations, they do not make the application of iPLA as an indicator in environmental health unfeasible. On the contrary, evidencing the risk to human health resulting from the presence of pesticide residues in drinking water provides important subsidies to guide intersectoral public policies. Based on the iPLA results, we can direct specific investments to promote and protect the health of the population in more vulnerable areas. The iPLA proposal is unique and offers a versatile indicator, with replication potential on a global scale, as its equation can be adapted according to local MPV parameters.

Thus, in this research, we contribute to the development of a practical, accessible, and relevant indicator for environmental health surveillance, able to evaluate the health risk of the population exposed to pesticide residues in drinking water, regardless of the type of chemical agent present.

## References

[1] Augusto LGS (2003). Saúde e vigilância ambiental: um tema em construção. Epidemiol Serv Saúde.

[2] Melanda VS, Galiciolli MEA, Lima LS, Figueiredo BC, Oliveira CS (2022). Impact of pesticides on cancer and congenital malformation: a systematic review. Toxics.

[3] Embrandiri A, Singh RP, Ibrahim HM, Khan AB (2012). An epidemiological study on the health effects of Endosulfan spraying on Cashew plantations in Kasaragod district, Kerala, India. Asian J Epidemiol.

[4] Lee J, Jang H, Pearce EN, Shin HM (2025). Exposome-wide association study of thyroid function using U.S. National Health and Nutrition Examination Survey data. Environ Res.

[5] Cao L, Kang Q, Tian Y (2024). Pesticide residues: Bridging the gap between environmental exposure and chronic disease through omics. Ecotoxicol Environ Saf.

[6] Brasil. Ministério da Saúde (1975). Portaria nº 635/BSB, de 26 de dezembro de 1975.

[7] Brasil. Ministério da Saúde (2011). Portaria nº 2.914, de 12 de dezembro de 2011.

[8] Brasil. Ministério da Saúde (2021). Portaria GM/MS nº 888, de 4 de maio de 2021.

[9] Brasil. Ministério da Saúde (2020). Manual do Sistema de Informação de Vigilância da Qualidade da Água para Consumo Humano - Sisagua: perfil empresa (prestadores de serviços de abastecimento de água).

[10] Brasil. Ministério da Saúde (2021). SISAGUA Treinamento.

[11] Bernal E, Guo X (2014). Advances in gas chromatography.

[12] Oliveira MLC, Faria SC (2008). Indicadores de saúde ambiental na formulação e avaliação de políticas de desenvolvimento sustentável. Rev Bras Ciências Ambient.

[13] R Core Team (2016). R: A language and environment for statistical computing.

[14] Ibañez HC, Figueiredo BC de (2010). Certificado de Registro de Patente do Geomedicina.

[15] Brasil. Ministério da Saúde. Secretaria de Vigilância em Saúde. Departamento de Saúde Ambiental do Trabalhador e Vigilância das Emergências em Saúde Pública (2020). Manual do Sistema de Informação de Vigilância da Qualidade da Água para Consumo Humano - Sisagua.

[16] Boutsidis C, Mahoney MW, Drineas P (2008). Unsupervised feature selection for principal components analysis.

[17] Organização Pan-Americana da Saúde (OPAS) (2018). Indicadores de Saúde: elementos conceituais e práticos.

[18] Brasil. Ministério da Saúde (2002). Textos de epidemiologia para vigilância ambiental em saúde.

[19] Secretaria de Estado da Agricultura e do Abastecimento do Paraná (2024). Inovação e tecnologia são fundamentais para agronegócio do futuro, aponta secretário.

[20] European Environment Agency (2024). Pesticidas em rios, lagos e águas subterrâneas na Europa.

[21] Agência Estadual de Notícias do (2024). Governo do Paraná busca novas tecnologias em irrigação para agricultura no Nebraska.

[22] Instituto Paranaense de Desenvolvimento Econômico e Social (2024). Portal.

[23] Pereira LAS, Oliveira AKM, Galafassi C (2023). Análise da associação entre a produtividade e do consumo de agrotóxicos em lavouras de algodão nos principais estados produtores. Obs Econ Latinoam.

[24] Fundação Heinrich Böll (2023). Atlas dos agrotóxicos: fatos e dados do uso dessas substâncias na agricultura.

[25] Carla S, Panis C, Zanetti L, Gurzenda S, Cruz J, Castro M (2022). Widespread pesticide contamination of drinking water and impact on cancer risk in Brazil. Environ Int.

[26] Oliveira DC, Lotufo J, Carvalho B (2019). Sistema de Informação de Vigilância da Qualidade da Água para Consumo Humano (Sisagua): características, evolução e aplicabilidade. Epidemiol Serv Saúde.

