# Supplementary material for: Pesticide threshold weighing indicator: application in the State of Paraná, Brazil
Source: Rev Bras Epidemiol. 2025 Aug 8;28:e250045. doi: 10.1590/1980-549720250045 (PMC12333892; doi:10.1590/1980-549720250045)
Supplement: Supplementary file 1 [file 1980-5497-rbepid-28-e250045-sup.pdf]

Tabela suplementar 1 Relação de Agrotóxicos Detectados e Analisados em 393 Municípios do Paraná, 2014-2018.

Item	Nome Agrotóxico	Item	Nome Agrotóxico
1	2,4 D + 2,4,5 T	15	Lindano
2	Alaclor	16	Mancozebe
3	Aldicarbi	17	Mtamidofós
4	Aldrin	18	Metolacloro
5	Atrazina	19	Molinato
6	Carbedazin +-benomil	20	Parationa Metilica
7	Carbofurano	21	Pendimetalina
8	Clordano	22	Permetrina
9	Clorpirifós+clopirifós oxon	23	Profenofós
10	DDD+DDE+DDT	24	Simazina
11	Diuron	25	Terbuconazol
12	Endossulfan	26	Terbufós
13	ENdrin	27	Trifruralina
14	Glifosato+ AMPA		

Fonte: a autora, 2022

Figura Suplementar 1 –Correlação entre a Produtividade (kg/ha) e os Registros de Agrotóxicos na Água Potável, segundo o iPLA, Paraná, 2014–2018.

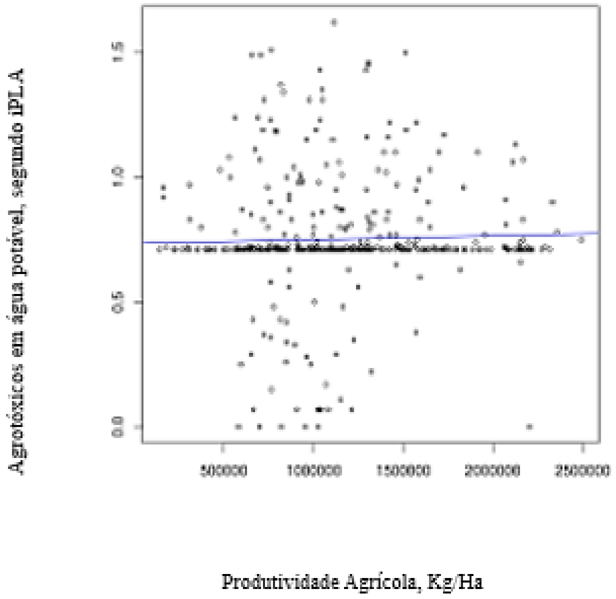

Fonte: a autora, 2022, obtido com o Geomedicina®.
