# Supplementary material for: Pesticide threshold weighing indicator: application in the State of Paraná, Brazil
Source: Rev Bras Epidemiol. 2025 Aug 8;28:e250045. doi: 10.1590/1980-549720250045 (PMC12333892; doi:10.1590/1980-549720250045)
Supplement: Translation

# Translation: Indicador de ponderação de limiares de agrotóxicos: aplicação no estado do Paraná

**DOI:** 10.1590/1980-549720250045.2

**Authors:** Viviane Serra Melanda, Humberto Cereser Ibañez, Henrique Aparecido Laureano, Luíza Siqueira Lima, Bonald Cavalcante Figueiredo, Cláudia Sirlene Oliveira

**Affiliations:** I Instituto de Pesquisa Pelé Pequeno Príncipe - Curitiba (PR), Brasil.; II Faculdades Pequeno Príncipe - Curitiba (PR), Brasil.; III Secretaria de Estado da Saúde do Paraná - Curitiba (PR), Brasil.

**Keywords:** Indicadores básicos de saúde, Agroquímicos, Epidemiologia, Poluentes da água

## Abstract

**Objetivo:**

Analisar os dados do Sistema de Informação da Vigilância da Qualidade da Água para Consumo Humano (Sisagua) para desenvolver um indicador de saúde ambiental que avalie o risco de resíduos de agrotóxicos na água potável.

**Métodos:**

Trata-se de um estudo epidemiológico ecológico, com dados retrospectivos. Foram analisadas informações sobre o limite de detecção (LD), limite de quantificação (LQ) e valor máximo permitido (VMP) de resíduos de agrotóxicos em água potável registradas pelos municípios do estado do Paraná no período de 2014 a 2020. Foram aplicadas abordagens de estatística descritiva e inferencial, incluindo testes de associação, correlação, hipóteses e probabilidade, por meio de análises espaciais e temporais.

**Resultados:**

Foi desenvolvido um indicador de ponderação de limiar de agrotóxico (iPLA), com capacidade explicativa de variabilidade superior a 77%. A variável atribuível de risco está relacionada principalmente ao VMP, que recebeu maior peso, enquanto o LD e o LQ foram atribuídos com menor peso.

**Conclusão:**

O iPLA demonstrou capacidade de representar os níveis de concentração de agrotóxicos na água potável. As categorias de risco definidas pelo indicador - controlado, silencioso e atenção - configuram-se uma ferramenta de grande utilidade para a vigilância em saúde, pois possibilitam o reconhecimento do nível de risco da água potável local à saúde humana. Além disso, o iPLA permite que a gestão pública implemente ações de controle e melhoria da qualidade da água para consumo humano.

## INTRODUÇÃO

A avaliação do risco dos agrotóxicos é fundamental, visto que estes estão associados a efeitos adversos sobre funções orgânicas vitais^1^ ^,^ ^2^ ^,^ ^3^ ^,^ ^4^ ^,^ ^5^. Embora no Brasil os padrões de potabilidade da água tenham sido oficialmente orientados pela Portaria MS/GM nº 635/1975^6^, apenas em 2011, com a publicação da Portaria MS/GM nº 2.914/2011, foram instituídos os procedimentos de controle e vigilância da qualidade da água para consumo humano. A Portaria MS/GM nº 2.914/2011 estabelece padrões de qualidade para potabilidade da água, indicando substâncias químicas que representam risco à saúde. Essas substâncias estão agrupadas em quatro categorias: inorgânicas, orgânicas, agrotóxicos e desinfetantes e produtos secundários da desinfecção^7^. Tais padrões foram atualizados em 2021, com a publicação da Portaria MS/GM nº 888^8^.

Concomitantemente, o sistema de informação oficial disponibilizado pelo Ministério da Saúde para inserção de dados provenientes do monitoramento da água de consumo humano no Brasil, o Sisagua, recebeu atualizações, visando ao melhor controle de inconsistências na entrada de dados, além de melhorias arquitetônicas para facilitar seu uso^9^. O Sisagua faz parte do Programa Nacional de Vigilância da Qualidade da Água para Consumo Humano (Vigiagua), que auxilia no gerenciamento dos riscos à saúde associados à qualidade da água para consumo humano^10^. Os municípios são os responsáveis por alimentar o Sisagua com o registro dos resultados obtidos nas análises de potabilidade da água.

Apesar da importância dos padrões de exposição a agrotóxicos definidos por órgãos reguladores, há barreiras técnicas quanto aos valores de referência usados na validação de métodos analíticos^11^. A existência de tais dificuldades pode prejudicar ou atrasar o reconhecimento de padrões de risco à saúde da população exposta aos agrotóxicos.

Os indicadores de saúde ambiental podem subsidiar as estratégias de prevenção, controle de riscos e promoção da saúde, bem como o monitoramento dos resultados epidemiológicos dessa relação^12^, no entanto não foi identificado na literatura científica um modelo de indicador que aponte a relação entre a presença de resíduo de agrotóxico em água potável e o potencial risco à saúde humana.

Com o propósito de trazer conhecimento sobre os riscos decorrentes da presença de resíduos de agrotóxicos em água potável para a saúde humana, este estudo desenvolveu um indicador de ponderação de limiar de agrotóxicos (iPLA). O objetivo foi simplificar o monitoramento e a classificação de riscos, possibilitando mais agilidade no desenvolvimento de políticas de saúde e estratégias intersetoriais envolvendo meio ambiente e saúde pública.

## MÉTODOS

Este é um estudo epidemiológico transversal, baseado em dados retrospectivos, com análise estatística descritiva e inferencial. As análises inferenciais incluem testes de associação, correlação e hipóteses, além da avaliação de probabilidades por meio de análises espaciais e temporais. Para as análises estatísticas, foram utilizados os *softwares* R^13^ e Geomedicina^14^. Este estudo foi aprovado pelo Comitê de Ética em Pesquisa da Faculdades Pequeno Príncipe (Certificado de Apresentação de Apreciação Ética nº 29734420.9.0000.5580), bem como pelo Comitê de Ética em Pesquisa da Secretaria de Estado da Saúde do Paraná (Certificado de Apresentação de Apreciação Ética nº 4.000.930/29734420.9.3002.5225), sediado no Hospital do Trabalhador.

Foi desenvolvido um indicador para avaliar o risco da presença de resíduos de agrotóxicos na água potável. Para isso, foram utilizados dados secundários provenientes de todos os registros disponíveis no Sisagua referentes ao estado do Paraná, na série histórica de 2014 a 2020. As amostras do Sisagua são coletadas nos municípios, em águas superficiais distribuídas pelos sistemas de abastecimento de água e/ou por soluções alternativas coletivas^15^.

Os dados analisados são de domínio público, disponibilizados pelo Ministério da Saúde e obtidos por *download* direto no ambiente virtual do governo federal, no conjunto de dados abertos do Sisagua, opção “Controle Semestral”. Foram incluídos na análise todos os municípios que apresentaram pelo menos um registro no grupo de parâmetros de agrotóxicos.

Ao tratar o banco de dados, observou-se que os resultados continham tanto registros numéricos (em mg/L ou μg/L) quanto nominais (MENOR_LD ou MENOR_LQ). Os campos de registro dos limites de detecção (LD) e de quantificação (LQ) de cada estação de tratamento de água apresentavam variações significativas. Para integrar dados nominais e numéricos, aplicamos contagens desses registros de quantificação, de tal forma que tanto os resultados numéricos como os nominais puderam ser considerados de forma equivalente.

Os valores nominais foram atribuídos às seguintes classes:

- menor que o LD (<LD);
- menor que o LQ (<LQ).

Os valores numéricos foram atribuídos às seguintes classes:

- entre o LQ e o valor máximo permitido (≥LQ e <VMP);
- maior ou igual ao VMP (≥VMP).

Dessa forma, após contabilizar todas as ocorrências registradas nos municípios em uma das quatro faixas, aplicou-se a contagem de peso obtido da análise de componentes principais (do inglês, *principal component analysis*-PCA), que corroborou com a identificação da combinação linear ótima das variáveis que melhor explicam a variabilidade dos dados.

A eliminação de inconsistências nos valores registrados no Sisagua foi realizada da seguinte maneira:

- Valores numéricos iguais a 0 foram atribuídos à classe <LD;
- Dos valores numéricos superiores ao VMP, foram excluídos os registros cujo Z-*score* [(valor registrado - média de todos os valores)/desvio padrão] fosse superior a três vezes o VMP.

### Declaração de disponibilidade de dados

Declaração de disponibilidade de dados: Todos os conjuntos de dados foram gerados ou analisados no estudo atual.

## RESULTADOS

### Desenvolvimento do indicador de ponderação de limiar de agrotóxico

Esse processo envolveu a leitura e a interpretação circunstancial dos dados, considerando as seguintes críticas:

- Crítica 1: delineamento das variáveis.

- <LD;
- <LQ;
- <VMP;
- ≥VMP.

- Crítica 2: atribuição de pesos às variáveis.

O peso de cada variável foi determinado pela PCA, consideradas as medições de LD, LQ e VMP como independentes e potencialmente correlacionadas no espaço (Tabela 1). Para melhor acomodação da variabilidade, os resultados foram normalizados, permitindo verificar que os quatro componentes, em conjunto, explicaram 100% da variabilidade das medidas. Observou-se que o PCA1 (≥VMP) explicou 77% da variabilidade total, enquanto os PCA2, PCA3 e PCA4, juntos, representaram os 23% restantes (Figura 1). Como o primeiro componente principal (PCA1) explicou por si só 77% da variabilidade total, ele foi selecionado para a construção do indicador PCA, garantindo a preservação da maior parte da informação relevante. Com base nisso, realizou-se um estudo comparativo da variabilidade estatística para verificar a correspondência entre os componentes principais e os resultados obtidos nas amostras (Tabela 2).

**Tabela 1.** Variabilidade da quantidade de registros (qt.reg) por vetor LD, LQ e VMP, Paraná, 2014-2020.

Indicador	<LD	<LQ	<VMP	>VMP
Primeiro autovetor (pesos)	0,16021	0,98707	0,00247	-0,00179
PCA (qt.reg)	0,07	0,71	1,43	2,14

LD: limite de detecção; LQ: limite de quantificação; VMP: valor máximo permitido; PCA: análise de componentes principais.

**Tabela 2.** Medidas descritivas da distribuição dos argumentos analisados referentes aos resultados dos registros de agrotóxicos em água potável nos municípios do Paraná, 2014-2020.

	>LD	>LQ	>VMP	≥VMP
Mínimo	0	0	0	0
Primeiro quartil	80	214	1	0
Mediana	109	312	10	0
Média	169,85	462,52	46,84	10,26
Terceiro quartil	190,5	566,0	46,0	6,5
Máximo	3937	5344	2382	225
Desvio padrão	251,06	507,81	142,14	24,99
Moran	0,0110367	0,110101343	0,0634899	0,1443155
Valor esperado	-0,0025216	-0,0025126	-0,0025126	-0,0025156
Variância	0,0005701	0,0009257	0,0007488	0,0009084
Valor-p	0,570383	0,000214	0,015862	0,000011

LD: limite de detecção; LQ: limite de quantificação; VMP: valor máximo permitido.

**Figura 1.** Análise da variância percentual e percentual acumulado, por componente principal de agrotóxico em água potável nos municípios do Paraná, 2014-2020.

PCA: análise de componentes principais.

A robustez do modelo foi avaliada indiretamente pela proporção da variabilidade explicada pelo PCA1, indicando sua adequação para representar os dados sem a necessidade de componentes adicionais. Além disso, como a PCA é um método não supervisionado, sua validação tradicional por técnicas como validação cruzada não se aplica diretamente, pois não há um conjunto de treinamento e teste a ser comparado^16^, no entanto verificamos a estabilidade das cargas fatoriais (pesos) e sua coerência interpretativa, garantindo que a construção do indicador fosse consistente com a estrutura dos dados.

- Crítica 3: desenvolvimento do iPLA.

Como o número de registros pode variar em cada série temporal, foi criado o iPLA com o objetivo de possibilitar a comparabilidade de novos cenários. Para atribuir maior peso às contagens de resultados das análises de água potável que se enquadraram nas faixas definidas em relação ao VMP (>LQ e <VMP; e ≥VMP), aplicaram-se as seguintes constantes, baseadas nos valores do PCA (Tabela 1):

- Para resultados na faixa ≤LD: 0,07;
- Para resultados na faixa >LD e ≤LQ: 0,71;
- Para resultados na faixa >LQ e <VMP: 1,43;
- Para resultados na faixa ≥VMP: 2,14;

O cálculo do iPLA é representado pela Equação 1:

iPLA = menor_ld/qt_result*0,07 + menor_lq/qt_result*0,71 + menor_vmp/qt_result*1,43 + maigu_vmp/qt_result*2,14 (1)

Em que:

menor_ld: a contagem dos resultados de análise de determinado agrotóxico, nas estações de tratamento de água de determinado município, cujos valores foram ≤LD;
qt_result: a quantidade total de resultados referentes a determinado agrotóxico obtidos nas estações de tratamento de água de determinado município durante o período analisado;
menor_lq: a contagem dos resultados de análise de determinado agrotóxico, nas estações de tratamento de água de determinado município, cujos valores foram >LD e ≤LQ;
menor_vmp: a contagem dos resultados de análise de determinado agrotóxico, nas estações de tratamento de água de determinado município, cujos valores foram >LQ e <VMP;
maigu_vmp: a contagem dos resultados de análise de determinado agrotóxico, nas estações de tratamento de água de determinado município, cujos valores foram ≥VMP;
0,07: a constante aplicada à contagem de resultados com valores ≤LD;
0,71: a constante aplicada à contagem de resultados com valores >LD e ≤LQ;
1,43: a constante aplicada à contagem de resultados com valores >LQ e <VMP;
2,14: a constante aplicada à contagem de resultados com valores ≥VMP.

Por conseguinte, a ponderação dos limiares de agrotóxicos estabeleceu constantes que atribuíram maior peso ao indicador do município nos casos em que os resultados estavam próximos (constante=1,43) ou superiores (constante=2,14) ao VMP. Em contrapartida, quando os resultados predominaram entre os limites de detecção e quantificação, as constantes atribuídas - respectivamente, 0,07 e 0,71 - conferiram menor peso ao indicador.

O modelo adotado na construção do iPLA validou a proposta do indicador de saúde ambiental para a análise da concentração de agrotóxicos em água potável, uma vez que apresentou resultados estatisticamente comprovados e representativos da realidade. Para interpretar os resultados do iPLA, foram definidos três grupos com base nas variações em torno das categorias LD, LQ e VMP, os quais receberam classificações em níveis de risco (Tabela 3). A definição do nível de risco atribuído a cada grupo foi estabelecida com base nos limiares das variáveis relacionadas às respectivas constantes. Assim, o iPLA possibilitou uma interpretação objetiva do risco associado à presença de agrotóxicos na água destinada ao consumo humano.

- Crítica 4: PCA *versus* iPLA.

Foram comparadas as medidas descritivas do iPLA e do PCA, sendo possível observar que o iPLA apresentou ótima capacidade de representar a contaminação da água potável. Ele teve desvio padrão menor que o do indicador PCA e utilizou pesos dinâmicos, ou seja, que variaram conforme a quantidade de registros, permitindo a equivalência dos resultados obtidos quando utilizado o indicador em diferentes localidades (Tabela 4).

**Tabela 3.** Categorias de risco iPLA em água potável.

Categoria	Variação do iPLA	Interpretação pelos percentuais das contagens nas classes <LD, <LQ, ≥LQ e <VMP e ≥VMP
Silencioso (I)	≥0 e <0,7	Localidade que não apresenta detecção de agrotóxicos e requer investigação quanto à qualidade das análises bioquímicas das amostras e dos dados inseridos no Sisagua.
Controlado (II)	≥0,7 e <1	Localidade que apresenta detecção de resíduos de agrotóxicos cujos valores não são quantificáveis e, portanto, são de risco não qualificável.
Atenção (III)	≥1	Localidade que apresenta detecção de resíduos de agrotóxicos cujos valores se qualificam para a existência de risco à saúde humana.

LD: limite de detecção; LQ: limite de quantificação; VMP: valor máximo permitido; iPLA: indicador de ponderação de limiar de agrotóxico; Sisagua: Sistema de Informação da Vigilância da Qualidade da Água para Consumo Humano.

**Tabela 4.** Análise descritiva dos indicadores de agrotóxicos em água potável, PCA e iPLA.

Indicador	Mínimo	Primeiro quartil	Mediana	Média	Terceiro quartil	Máximo	Desvio padrão
iPLA	0	0,53	0,58	0,62	0,68	1,51	0,2
PCA	-0,94	-0,5	-0,29	0	0,21	9,44	1

iPLA: indicador de ponderação de limiar de agrotóxico; PCA: análise de componentes principais.

Aplicação do indicador de ponderação de limiar de agrotóxico para análise situacional de risco epidemiológico no Paraná

Para análise epidemiológica espacial, optou-se por aplicar a série histórica de cinco anos (2014-2018), no estado do Paraná, a fim de verificar tendências nos resultados obtidos. Foram encontrados 27 tipos de resíduo de agrotóxicos nas amostras de água potável, segundo registros do Sisagua, em 393 dos 399 municípios do Paraná (Tabela Suplementar 1: https://drive.google.com/drive/folders/1bTyymbYahQXyZKlsn7felApqtyyrJneV?usp=sharing). A Tabela 5 apresenta a frequência e o percentual das análises registradas no Sisagua entre 2014-2018, considerando as variáveis LD, LQ e VPM. As contagens abrangem os 27 agrotóxicos analisados neste estudo, totalizando 176.533 registros. Destes, a maioria (82,2%) corresponde a valores nominais <LD.

**Tabela 5.** Frequência e percentual das análises de água registradas no Sisagua para as variáveis LD, LQ e VPM no estado do Paraná (2014-2018).

Ano	Número de registros <LD (%)	Número de registros <LQ (%)	Número de registros ≥LQ <VPM (%)	Número de registros ≥VPM (%)	Número total de registros
2014	2.567 (11,05)	19.663 (84,65)	625 (2,69)	374 (1,61)	23.229
2015	3.442 (10,90)	27.100 (85,82)	668 (2,12)	368 (1,17)	31.578
2016	2.512 (7,00)	32.090 (89,42)	806 (2,25)	478 (3,91)	35.886
2017	5.556 (13,05)	31.713 (74,48)	3.648 (8,57)	1.664 (3.91)	42.581
2018	1.830 (4,23)	34.547 (79,86)	5.872 (13,57)	1.010 (2.33)	43.259

LD: limite de detecção; LQ: limite de quantificação; VMP: valor máximo permitido; Sisagua: Sistema de Informação da Vigilância da Qualidade da Água para Consumo Humano.

Muitos municípios do Paraná apresentaram resultados de concentração de agrotóxicos nas amostras de água potável com valores <LQ, e alguns registraram amostras com concentrações acima dos valores permitidos. Além disso, observou-se o aumento no número de registros com valores ≥VMP ao longo dos anos. O risco associado ao nível de iPLA é especialmente elevado em municípios da região sudeste do Paraná, com destaque para o Vale do Ribeira, onde há municípios próximos ao estado de São Paulo e que se encontram no nível III de atenção. (Figura 2A).

**Figura 2.** Nível de risco de agrotóxico na água potável registrada pelo iPLA, por (a) Municípios e (b) densidade populacional, Paraná, 2014-2018.

iPLA: indicador de ponderação de limiar de agrotóxico.

Ao investigar se há correlação entre a produtividade (kg/ha) no Paraná, obtida do Sistema de Monitoramento do Comércio e Uso de Agrotóxicos do Paraná (Siagro), e o iPLA, os resultados não indicaram correlação entre esses indicadores (Figura Suplementar 1: https://drive.google.com/drive/folders/1bTyymbYahQXyZKlsn7felApqtyyrJneV?usp=sharing). Já a análise da distribuição geográfica do risco de agrotóxicos (iPLA) por densidade populacional revelou que as regiões sul, sudeste e sudoeste do estado do Paraná se destacam com os maiores índices (Figura 2B). De acordo com o iPLA, a maior parte dos municípios do Paraná apresenta risco nível III, o que indica uma condição de saúde insegura associada à presença de agrotóxicos na água potável.

## DISCUSSÃO

Em 1998, a Organização Mundial da Saúde (OMS) estabeleceu critérios para o desenvolvimento de indicadores em saúde ambiental: aplicabilidade geral, solidez científica e utilidade para os usuários^17^ ^,^ ^18^. O iPLA foi desenvolvido com o objetivo de superar a barreira da aplicabilidade ao usuário, sendo estruturado para facilitar a interpretação dos resultados por meio de categorias de risco. Demonstrou-se sua aplicabilidade geral, uma vez que ele foi construído com base em resultados de amostras de água potável analisadas pelas redes de abastecimento dos municípios. A robustez científica do iPLA também foi evidenciada por meio de análises estatísticas que indicaram validade significativa em sua capacidade de representar o grau de contaminação por agrotóxicos na água potável. Quanto aos propósitos descritos pela OMS sobre o uso de indicadores para monitoramento da saúde^19^, o iPLA justifica-se por sua capacidade de descrever e avaliar riscos, bem como de promover a responsabilização, ao evidenciar a suscetibilidade da população exposta e revelar a real qualidade da água potável disponível à população.

Em publicação recente na Europa, foi constatada a importância do monitoramento em números absolutos de agrotóxicos em águas superficiais, com o objetivo de projetar padrões de qualidade para as águas subterrâneas. O estudo observou diferenças nos registros de monitoramento, como o número de amostras, de pesticidas monitorados e de locais analisados. Essas diferenças podem comprometer a interpretação das tendências no continente^20^. Em nosso estudo, o iPLA foi desenvolvido com base em dados registrados no banco de dados de um sistema de informação nacional e oficial do Brasil, o Sisagua, podendo, portanto, ser reproduzido em todas as unidades administrativas do país. Demonstrou-se estatisticamente que o iPLA tem capacidade de representar a contaminação da água por agrotóxico, sendo interpretado como um indicador negativo.

Tendo como característica a busca por inovação na agricultura, incluindo novas tecnologias de irrigação para o aumento da produtividade, o Paraná destaca-se como uma unidade federativa com intensa atividade de investimento e captação de recursos para o agronegócio, contribuindo significativamente para o crescimento da riqueza nacional^21^ ^,^ ^22^. Este estudo analisou a correlação entre a produtividade agrícola e a concentração de agrotóxicos na água potável do Paraná, por meio do iPLA, e não foi identificada correlação entre essas variáveis. Um estudo recente que investigou a relação entre produtividade agrícola e uso de agrotóxicos no Brasil tampouco apresentou resultados que permitam afirmar a existência de correlação entre essas duas variáveis exclusivamente^23^. Dessa forma, estudos que buscam analisar a correlação entre o uso de agrotóxicos e a produtividade precisam considerar os diversos elementos que coparticipam do manejo produtivo.

Embora o monitoramento da potabilidade da água seja uma prática amplamente adotada por governos em todo o mundo, a padronização dos métodos analíticos ainda representa um desafio a ser superado^11^. Persiste uma lacuna importante quanto à existência de um indicador de saúde capaz de classificar o nível de contaminação da água potável por resíduos de agrotóxicos que disponha de dados atualizados continuamente, tenha um cálculo viável e seja de fácil compreensão para os profissionais que atuam na vigilância em saúde ambiental. O iPLA é um indicador de saúde ambiental de aplicação prática, uma vez que seu monitoramento se baseia na frequência de registros no Sisagua, reunindo resultados nominais e quantitativos, transformando-os em uma variável de contagem inserida em uma equação composta de constantes predefinidas e consistentes. Os resultados obtidos são parametrizados em categorias de risco, o que torna sua análise simples e acessível.

No Brasil, a legislação permite níveis relativamente elevados de agrotóxicos na água potável e desconsidera a soma dos VMPs quando múltiplos resíduos estão presentes na mesma amostra. Além disso, os agrotóxicos não contemplados nas normativas vigentes não têm VMP estabelecido, o que dificulta a adoção de medidas de intervenção^23^. Mesmo quando os resultados das análises estão em conformidade com os limites estabelecidos, é imprescindível manter ações de vigilância em saúde sobre as populações potencialmente expostas. Há diferenças significativas nos VMP de agrotóxicos, tanto em alimentos quanto na água, entre os principais produtores mundiais, como Brasil, Estados Unidos, China, Japão, e também em relação às diretrizes da OMS. Os parâmetros definidos pelas normas regulamentadoras variam entre esses países. Por exemplo, na União Europeia, independentemente do ingrediente ativo, o VMP para água potável é de 0,0001 mg/L por substância, e o VPM para o somatório de todas as substâncias é de 0,0005 mg/L. Em contrapartida, no Brasil, Estados Unidos, China e Japão, cada agrotóxico tem um VMP individual regulamentado, sem a exigência de um limite máximo total para a soma dos resíduos presentes na mesma amostra^24^.

Nesse contexto, o desenvolvimento do iPLA se mostra altamente relevante, pois considera os registros iguais ou superiores ao VMP, independentemente do agente químico. Essa característica flexível revela-se estratégica na análise dos dados utilizados pelo indicador, uma vez que o iPLA permanece aplicável mesmo diante de alterações nos parâmetros normativos - como ocorreu no Brasil com a publicação da Portaria GM/MS nº 888/2021, que modificou os níveis de tolerância dos VMPs e alterou o grupo de agrotóxicos permitidos.

Em um estudo recente sobre a presença de agrotóxicos em amostras de água monitoradas pelo Vigiagua, foram analisadas 41.780 amostras. Os agrotóxicos mais frequentemente quantificados foram: atrazina, metolacloro, glifosato e 2,4-D. Menos de 10% das detecções ultrapassaram o limite de quantificação, no entanto um percentual elevado de substâncias foi identificado, embora não quantificável, o que possivelmente reflete limitações das técnicas analíticas utilizadas, e não necessariamente a ausência de risco^25^.

Nesse sentido, o iPLA, por apontar o risco circunstancial à saúde humana decorrente da exposição a agrotóxicos, configura-se como uma ferramenta de grande utilidade para a gestão pública. O indicador permite reconhecer o nível de risco à saúde da população exposta a qualquer agrotóxico presente na água potável, desde municípios categoria I, que são classificados como silenciosos e que merecem qualificação do monitoramento, municípios de categoria II, cujo monitoramento está sensível e os resultados não apontam para o risco à saúde humana, e municípios de categoria III, cujos resultados apontam para uma situação de atenção e alerta para vigilância em saúde.

Durante o desenvolvimento do iPLA, identificou-se uma limitação relevante relacionada à variabilidade dos métodos analíticos utilizados para mensurar os valores de LD, LQ e VMP, além das diferenças no controle de qualidade entre os laboratórios que analisam as amostras de água potável. Esses fatores podem gerar sub-registros ou variações na qualidade e na frequência dos dados registrados no sistema de informação oficial (Sisagua), cuja alimentação depende da capacitação técnica dos profissionais responsáveis. A possibilidade de entrada de dados nominais e numéricos caracteriza uma fragilidade de precisão no que diz respeito à parametrização dos resultados. Observou-se que, em 25% dos registros analisados, os resultados foram iguais ao VMP estabelecido para o agente químico. Essa regularidade estatística levanta a hipótese de inconsistências no banco de dados, pois os testes laboratoriais, por sua natureza, devem gerar uma ampla variedade de resultados, e não uma concentração tão significativa no valor exato do limite permitido.

Vale destacar que apenas os dados de controle da Vigilância em Saúde - como os relativos a desinfetantes residuais, turbidez, cor, pH, fluoreto, bactérias heterotróficas, coliformes totais e *Escherichia coli* - são transmitidos automaticamente. Os demais dados do Sisagua são inseridos manualmente por profissionais das secretarias municipais de Saúde ou pelas concessionárias de abastecimento de água para consumo humano^26^. Assim, reforça-se a importância da análise crítica e da qualificação contínua dos dados inseridos no sistema.

Apesar dessas limitações, elas não inviabilizam a aplicação do iPLA como indicador em saúde ambiental. Ao contrário, o reconhecimento do risco à saúde humana decorrente da presença de resíduos de agrotóxicos na água potável fornece subsídios importantes para orientar políticas públicas intersetoriais. Com base nos resultados do iPLA, é possível direcionar investimentos específicos para promoção e proteção da saúde da população em áreas mais vulneráveis. A proposta do iPLA é original e oferece um indicador versátil, com potencial de replicação em escala global, uma vez que sua equação pode ser adaptada conforme os parâmetros locais de VMP.

Dessa forma, esta pesquisa contribui para o desenvolvimento de um indicador prático, acessível e relevante para a vigilância em saúde ambiental, capaz de avaliar o risco à saúde da população exposta a resíduos de agrotóxicos na água potável, independentemente do tipo de agente químico presente.
